# A Highly Selective NIR Fluorescent Turn-on Probe for Hydroxyl Radical and Its Application in Living Cell Images

**DOI:** 10.3389/fchem.2019.00598

**Published:** 2019-08-28

**Authors:** Xingyu Qu, Wenting Song, Zhen Shen

**Affiliations:** ^1^State Key Laboratory of Coordination Chemistry, Nanjing National Laboratory of Microstructures, School of Chemistry and Chemical Engineering, Nanjing University, Nanjing, China; ^2^Department of Chemistry and Chemical Engineering, Jinzhong University, Jinzhong, China

**Keywords:** NIR fluorescent probe, hydroxyl radical, living cell images, energy transfer, BODIPY

## Abstract

A highly selective NIR fluorescent turn-on probe for hydroxyl radical (·OH) has been built up using triphenylphosphine as a reactive-site for ·OH in an energy transfer cassette **2b** consisting of 8-2′-(thiophen-2-yl) quinoline (**TQ**) as a donor and 3,5-diphenylphosphinostyryl-substituted BODIPY as an acceptor, which exhibits ca. 317 nm pseudo Stokes' shift due to efficient through-bond energy transfer (up to 169%). The triphenylphosphine substituent of **2b** selectively oxidized by ·OH over the other reactive oxygen species (ROS) and the reactive nitrogen species (RNS) resulting in fluorescence enhancement in aqueous solution and in living cells.

## Introduction

Free radicals that are natrually produced *in vivo*, by normal cellular metabolism or through disease process and xenobiotic activities, often cause many of the tissue changes associated with toxicities and disease processes (Dixon and Stockwell, 2014). The hydroxyl radical (·OH) is the most reactive species of oxygen in biological systems. It has a half-life about 1 ns and reacts unselectively in preferences for coreactants, resulting in a wide range of initial molecular changes, such as oxidative damage to DNA, proteins, lipids, and mediate redox alteration of cell-membrane Ca^2+^ channels (Cleveland and Kastan, 2000; Ayala et al., 2014). The difficulty in detecting such a short-lived species has made determining its involvement in toxic events difficult. Therefore, developing a rapid and sensitive method for monitoring ·OH in biological systems greatly improves our understanding of the roles of this reactive species in toxic mechanisms and disease processes (Wiseman and Halliwell, 1996; Pennathur et al., 2001). The common detection method for ·OH is the electron spin resonance (ESR). As the ESR measures the electron paramagnetic resonance spectrum of a spin adduct derivative after spin trapping, this method is insensitive and only qualitative estimates of ·OH (Valavanidis, 2000; Vidrio et al., 2008). Valavanidis to overcome these limitations, several fluorescent probes for ·OH have been developed. These probes include fluorescein with ·OH reactive-site (Zhang et al., 2016; Bai et al., 2017), cyanine dye based on a hybrid phenothiazine platform (Liu et al., 2016), a hybrid carbazole-cyanine dye (Zeng et al., 2017), fluorophore with nitroxide function group (Liras et al., 2016). However, limitation of these ·OH-responsive probes in intracellular imaging is their absorption and emission bands being situated in the Ultraviolet (UV) or Visible region, weak sensitivity or poor selectivity. Moreover, the difference in lifetimes of ROS/RNS further increases the difficulty to design multiple probes. Up to date, the approach of a single fluorescent probe to the simultaneous detections of several ROS/RNS has still been a challenging task.

The 4,4-Difluoro-4-bora-3*a*,4*a*-diaza-*s*-indacene (BODIPY) dyes have many favorable photophysical properties, such as high extinction coefficient, high fluorescence quantum yields, facile derivatization, and good photostability (Lu et al., 2014; Kowada et al., 2015). They have been investigated intensively as labeling reagents (Cheng et al., 2017), fluorescent switches (Dolan et al., 2017), chemosensor (Ren et al., 2018), and laser dyes (Zhu et al., 2018) in the last three decades. Since the absorption and emission bands of the unmodified BODIPY lie at *ca*. 500 nm, one important approach to red shift the main BODIPY absorption band is introducing styryl-substituents at 3-, 5- and/or 1-, 7-positions on the pyrrole moieties (Patalag et al., 2017; Verwilst et al., 2017). The following characteristics are highly desirable for intracellular imaging: (i) selectivity and sensitivity toward a specific ion; (ii) fluorescence maxima appear in the near infrared (NIR) region (650–900 nm); (iii) minimize the scattering effects from the excitation source (Ali et al., 2015, 2017). Herein, we report two NIR BODIPY probes using the triphenylphosphine as substituents at 3-, 5-positions of the BODIPY core. The two probes exhibits excellent optical properties and can be used as fluorescence turn-on chemosensor for ·OH, as ·OH -trigger oxidation of the triphenylphosphine. Their sensing properties have been investigated for living cell images. To the best of our knowledge, no attempt to employ NIR probe for detection of ·OH in living cells has previously been made.

## Materials and Methods

The ^1^H and ^13^C NMR spectroscopic measurements were carried out on a Bruker 500 MHz spectrometer. The measurements for ^1^H and ^13^C NMR were performed at 500 (DRX-500), and 125 MHz (DRX-500), respectively. Mass spectra were measured on a Bruker Daltonics Autoflex II^TM^ MALDI-TOF MS spectrometer. Fluorescence spectral measurements were carried out by using a Hitachi F-4600 fluorescence spectrophotometer. Electronic absorption spectra were recorded with a Shimadzu UV-2550 spectrophotometer. Cyclic voltammograms were recorded using a platinum working electrode, a platinum wire counter electrode and an Hg/Hg_2_Cl_2_ reference electrode. The measurements were carried out in dichloromethane (CH_2_Cl_2_) solution using 0.1 M Bu_4_NPF_6_ as the supporting electrolyte at a scan rate of 0.1 V/s. Peak potentials were determined from differential pulse voltammetry experiments. The Fc/Fc^+^ redox couple was used as an internal standard. Unless otherwise noted, all reagents or solvents were obtained from commercial suppliers and used without further purification. All air and moisture sensitive reactions were carried out under an argon atmosphere. Dry CH_2_Cl_2_ was obtained by refluxing and distilling over CaH_2_ under nitrogen. Dry THF was distilled from sodium/benzophenone.

X-ray crystallographic data for **ox-2a** were recorded at 100 K on a Rigaku CCD detector (Saturn 724) mounted on a Rigaku rotating anode X-ray generator (MicroMax-007HF) using Mo-Kα radiation from the corresponding set of confocal optics. The structure was solved by direct methods and refined on F^2^ by full-matrix least-squares using the Crystal Clear and SHELXS-2000 programs. CCDC 875597 contains the supplementary crystallographic data for this paper. These data can be obtained free of charge from The Cambridge Crystallographic Data Center via www.ccdc.cam.ac.uk/conts/retrieving.html (or from the Cambridge Crystallographic Data Center, 12, Union Road, Cambridge CB21EZ, UK; fax: (+44) 1223-336-033; email: deposit@ccdc.cam.ac.uk).

Spectra were measured in 1 cm quartz cuvettes with spectroscopic grade solvents. The slit width was set at 5 nm for both excitation and emission measurements. Cresyl violet perchlorate in methanol (Φ_f_ = 0.55) was used as the standard for the fluorescent quantum yield calculation using the absorption of the test sample. The emission spectra area was obtained from 550 to 800 nm. Dilute solutions (10^−6^ M) were used to minimize reabsorption effects. Fluorescence measurement were made three times for each dye and averaged. Quantum yields were determined using the following equation:

$$\begin{array}{l} \Phi_{\text{samp}}=\left(\Phi_{\text{stand}}\times \;{\text{F}}_{\text{samp}}/{\text{F}}_{\text{stand}}\right)\times \left({\text{A}}_{\text{samp}}/{\text{A}}_{\text{stand}}\right)\\ \;\times \left({{\text{n}}_{\text{samp}}}^{2}/{{\text{n}}_{\text{stand}}}^{2}\right) \end{array}$$

F_samp_ and F_samp_ are the quantum yield of the sample and the cresyl violet perchlorate standard. F_samp_ and F_stand_ are the areas under the emission spectra of the sample and standard. A_samp_ and A_stand_ are the absorbance values for the sample and standard at the excitation wavelength. n_samp_ and n_stand_ are the refractive index values of the solvents used for the sample and standard measurements. Molar extinction coefficients were obtained from the slope of a graph of absorbance vs. concentration for each dye at five different concentrations (10^−6^ M).

The HeLa cell line was provided by the Institute of Biochemistry and Cell Biology, SIBS, CAS (China). Cells were grown in high glucose Dulbecco's Modified Eagle Medium (DMEM, 4.5 g of glucose/L) supplemented with 10% fetal bovine serum (FBS) at 37°C and 5% CO_2_. Cells (5 × 10^8^/L) were plated on 14 mm glass coverslips and allowed to adhere for 24 h. Experiments to assess phorbol myristate acetate (PMA) uptake were performed over 2 h in the same medium.

Immediately before the experiments, cells were washed with PBS buffer and then incubated with 10 μM **2b** in PBS buffer for 2 h at 37°C. Cell imaging was then carried out after washing the cells with PBS buffer. Confocal fluorescence imaging was performed with a Zeiss LSM 710 laser scanning microscope and a 63 × oil-immersion objective lens. Cells incubated with **2b** were excited at 633 nm using a multi-line argon laser.

The compounds **1a** and **1b** were synthsised according to the literature (Qu et al., 2012). **1a** was obtained as yellow solid with 38.6% yield., ^1^H NMR(CDCl_3_, 500 MHz): 7.45 (*t, J* = 5 Hz, 3H), 7.27 (s, 2H), 6.95 (s, 2H), 2.53 (s, 6H), 1.34 (s, 6H).

**1b** was obtained as red solid with 65% yield. ^1^H NMR(CDCl_3_, 500 MHz): 8.19 (d, 1H, *J* = 10 Hz), 8.08 (d, 1H, *J* = 10.0 Hz), 7.81 (m, 2H), 7.76 (d, 1H, *J* = 3.5 Hz), 7.71 (t, 1H, *J* = 5.0 Hz), 7.51 (t, 1H, *J* = 5.0 Hz), 7.04 (d, 1H, *J* = 5.0 Hz), 6.02 (s, 2H), 2.57 (s, 6H), 1.76 (s, 6H).

The compounds **2a** and **2b** were synthsised according to the literature (Buyukcakir et al., 2009). Compound **1a** (0.16 mmol, 50 mg) and 2-(diphenylphosphino)benzaldehyde (0.32 mmol, 100 mg) were added to a 100 mL round bottomed flask containing 50 mL acetonitrile, and then piperidine (0.4 mL) and acetic acid (0.4 mL) were added to this solution. The mixture was heated under reflux by using a Dean Stark trap and reaction was monitored by TLC in solvent CH_2_Cl_2_. When all the starting material had been disappeared, the mixture were cooled to room temperature and concentrated at reduced pressure. The dark brown reaction mixture was washed with 100 mL water and extracted twice with 50 mL chloroform., dried over Na_2_SO_4_, and concentrated at reduced pressure. The crude products were purified by silica-gel column chromatography using mixture solvent (ethyl acetate:petroleum, 1:1, v/v) as the eluant to give **2a** as a black solid with 77% yield. ^1^H NMR (500 MHz, CDCl_3_): 8.04 (1 H, d, *J* = 5.1), 8.01 (1 H, d, *J* = 5.2), 7.98–7.92 (2 H, m), 7.65 (1 H, s), 7.62 (1 H, s), 7.47 (3 H, d, *J* = 3.4), 7.42 (2 H, t, *J* = 7.6), 7.33 (11 H, d, *J* = 2.8), 7.31–7.27 (7 H, m), 7.21 (2 H, t, *J* = 7.5), 6.93–6.86 (2 H, m), 6.45 (2 H, s), 1.38 (6 H, s); ^14^B NMR (160 MHz, CDCl_3_): 1.05; ^31^P NMR (202 MHz, CDCl_3_): −14.29. MALDI-TOF MS: m/z: 867.6 (M^+^) (Figures S1, S2 and S9, ESI).

Compound **1b** (0.11 mmol, 50 mg) and 2-(diphenylphosphino)benzaldehyde (0.33 mmol, 110 mg) were added to a 100 mL round bottomed flask containing 60 mL mixture solvent (acetonitrile: 1,2-dichloroethane, 1:1, v/v), and then piperidine (0.4 mL) and acetic acid (0.4 mL) were added to this solution. The mixture was heated under reflux by using a Dean Stark trap and reaction was monitored by thin-layer chromatography (TLC) in solvent CH_2_Cl_2_. When all the starting material had been disappeared, the mixture were cooled to room temperature and concentrated at reduced pressure. The dark brown reaction mixture was washed with 100 mL water and extracted twice with 50 mL chloroform., dried over Na_2_SO_4_, and concentrated at reduced pressure. The crude products were purified by silica-gel column chromatography using mixture solvent (dichloromethane:petroleum, 1:1, v/v) as the eluant to give **2b** as a black solid with 45% yield. ^1^H NMR (500 MHz, CDCl_3_) 8.23–8.16 (2 H, m), 8.05 (3 H, dd, *J* = 15.9, 5.4), 7.99–7.93 (2 H, m), 7.82 (3 H, dd, *J* = 12.3, 8.3), 7.75–7.69 (2 H, m), 7.63 (2 H, d, *J* = 15.7), 7.55–7.49 (3 H, m), 7.44 (3 H, dd, *J* = 17.4, 9.7), 7.33 (12 H, d, *J* = 2.3), 7.29 (7 H, dd, *J* = 7.3, 2.4), 7.22 (2 H, t, *J* = 7.6), 7.06–7.00 (1 H, m), 6.90 (3 H, dd, *J* = 7.3, 4.9), 6.48 (2 H, s), 1.76 (6 H, s) 13C NMR (126 MHz, CDCl3) δ = 153.17, 151.57, 142.34, 140.90, 140.74, 136.85, 136.70, 136.59, 136.23, 135.05, 134.83, 134.05, 133.90, 133.68, 132.38, 131.99, 130.10, 129.34, 129.25, 128.88, 128.67, 128.63, 127.52, 127.40, 126.45, 125.78, 120.45, 118.51, 117.26, 77.27, 77.02, 76.77, 14.16, −0.00.^31^P NMR (202 MHz, CDCl_3_): −14.30. MALDI- TOF MS: m/z: 1002 (M^+^) (Figures S3–S5 and S10, ESI).

The compound **ox-2a** was synthsized according to the literature (Ali et al., 2017). Compound **2a** (0.02 mmol) and hydrogen peroxide (0.2 mmol) were added to a 100 mL round bottomed flask containing 50 mL tetrahydrofuran, and then FeSO_4_•7H_2_O (0.12 mmol) were added to this solution. The solution was stirred for 30 min at ambient temperature. When all the starting material had disolved, was washed with 100 mL water and extracted twice with 50 mL chloroform., dried over Na_2_SO_4_, and concentrated at reduced pressure. The products were the remainder that the mixture was washed with 100 mL of water to give **ox-2a** as a black solid in quantitative yield. ^1^H NMR (500 MHz, CDCl_3_): 8.13 (2 H, d, *J* = 16.1), 8.03 (2 H, dd, *J* = 7.7, 3.9), 7.67 (8 H, dd, *J* = 11.8, 7.5), 7.63–7.41 (20 H, m), 7.17 (2 H, dd, *J* = 14.1, 7.6), 6.28 (2 H, s), 1.33 (6 H, s); ^13^C NMR (126 MHz, CDCl_3_): δ 152.42, 142.30, 141.26, 139.50, 134.90, 134.31, 133.81, 133.71, 133.57, 133.23, 132.40, 132.31, 131.97, 131.90, 131.02, 130.22, 129.06, 128.69, 128.59, 128.25, 127.69, 127.58, 121.42, 118.64, 77.26, 77.01, 76.75, 14.47. ^14^B NMR (160 MHz, CDCl_3_): 0.95; ^31^P NMR (202 MHz, CDCl_3_): 31.41. MALDI- TOF MS: m/z: 800 (M^+^-F) (Figures S6–S8 and S11, ESI).

## Results and Discussions

### Design and Synthesis

**Scheme 1:**
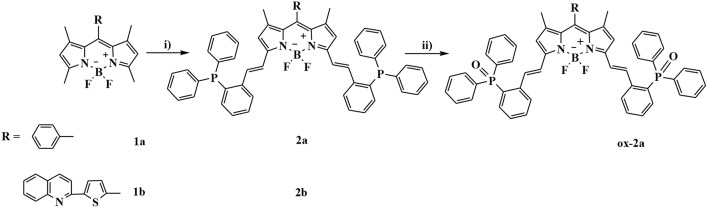
Synthetic procedures for compounds **2a** and **2b**: (i) 2-diphenylphosphinobenzaldehyde, AcOH/piperidine, in CH_3_CN, reflux, 77% for **2a** and 45% for **2b**; (ii) Fenton's reagent, in THF, rt, 100%.

The synthesis of BODIPYs **2a** and **2b** bearing triphenylphosphine as a reactive-site for ·OH are outlined in Scheme 1. Compounds **1a** and **1b** were synthesized in 65 and 39% yields according to a published procedure. Using knoevenagel condensation method, the sensors **2a** and **2b** can be obtained in 77% and 45% yield by condensation of **1a** and **1b** with 2-diphenylphosphinobenzaldehyde. Oxidation reaction of **2a** with hydroxyl radical gives compound **ox-2a** in 100% yield.

**Figure 1:**
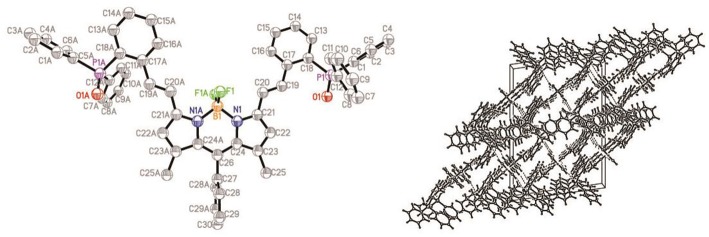
ORTEP views of the molecular structure of **ox-2a** with the thermal ellipsoids set at 30% probability **(left)** and packing diagram **(right)**.

The structure of compound **ox-2a** has been determined by X-ray analysis. Single crystal of **ox-2a** is obtained by slow evaporation of hexane/CH_2_Cl_2_ solution at ambient temperature. Similar to the previously reported structures of alkyl substituted BODIPYs (Qu et al., 2012), the *meso-*phenyl ring is virtually orthogonal to the indacene plane with the torsion angle being 87.69°. The indacene plane of BODIPY is nearly planar with the deviations from the mean plane 0.0285 Å. It is interesting to note that the dihedral angles between the neighboring phenyl in triphenylphosphine are 78.53°, 88.57°, 88.56°, respectively in the unit cell (Figure 1 and Table S1).

### Spectral Properties

**Figure 2:**
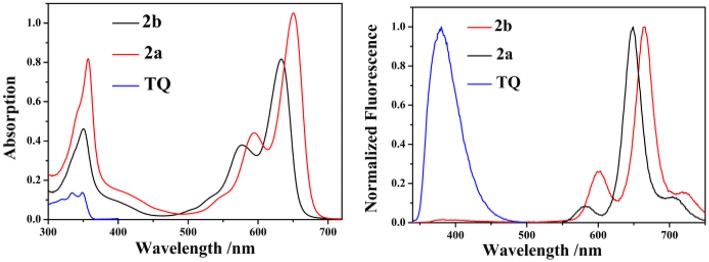
The absorption **(left)** and emission **(right)** spectra of **2a**, **2b**, and **TQ** in CH_2_Cl_2_ (10 μM, λ_ex_ = 334 nm for **2b** and **TQ**, λ_ex_ = 555 nm for **2a**).

The UV/vis absorption and emission spectra of **2a**, **2b** and **TQ** are measured in various solvents with different polarities and the photophysical properties are summarized in Table 1. As shown in Figure 2, the absorption maxima of **2a** and **2b** are centered at 628 and 650 nm, respectively, which can be ascribed to the S_0_ → S_1_ transition of the BODIPY. The absorption band at 350 nm for **2a** and **2b** can be assigned to the intramolecular charge transfer (ICT) band due to the electron-donating 2-diphenylphosphinostyryl moiety. The absorption spectra of **2a**, **2b**, and **ox-2a** are slightly varied with increasing the solvent polarity (Table S2, ESI). Upon exciting 2-(thiophen-2-yl)quinoline moiety at 334 nm in **2b**, the emission from the **TQ** moiety is almost quenched completely, instead strong emission at 665 nm is observed. These results imply that efficient energy transfer from the donor to the BODIPY acceptor occurs. The energy transfer efficiency is evaluated according to the equation: [1-I_d_]/I_p_ × 100%, where I_d_ is the fluorescence intensity of **2b** excited at 334 nm, I_p_ is the fluorescence intensity of **2b** excited at 580 nm (Yan et al., 2016). In addition, due to the antenna effect (Greene et al., 2017), the fluorescence quantum yields of **2b** upon excitation of the **TQ** moiety (λ_ex_ = 334 nm) are higher than that obtained by directly excitation of the BODIPY acceptor (λ_ex_ = 580 nm) (Table 1). The fluorescence quantum yield of **2b** in DMSO (λ_ex_ = 580 nm; Φ = 0.08) is weak, which can be ascribed to the photo-induced electron transfer (PET) from the triarylphosphine moiety to the BODIPY fluorophore (Table S2, ESI).

**Table 1:** Optical properties of **2a**, **2b**, and **TQ** in CH_2_Cl_2_ at 298 K.

**compounds**	$\lambda_{\text{abs}}\max$ (nm)/log**ε_max_**	**λ_flu_ (nm)**	**Quasi-Stokes' shift [cm^**−1**^]**	$\phi^{\text{b}}{}_{\text{f}}$**ex334**	$\phi^{\text{a}}{}_{\text{f}}$ **accptor**	**ETE^b^**	**τ(ns)^c^**
**2a**	633/4.91, 577/4.58, 350/4.65	650	55,555	–	0.72	–	4.25
**2b**	650/5.05, 594/4.64, 350/4.65,	665	3,154	0.54	0.32	169%	4.03
**TQ**	334/4.27	386	19,230	0.09	–	–	–

^a^Fluorescence quantum yield.

b Energy transfer efficiency.

c *Fluorescence lifetimes*.

### Fluorescence Detection of ·OH

**Figure 3:**
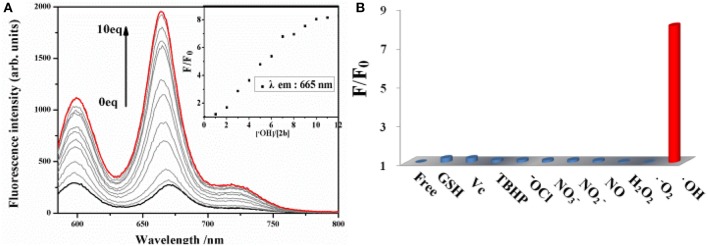
**(A)** Changes in the fluorescence spectrum of **2b** (10 μM in DMSO) as the concentration of ·OH is increased upon excitation at 580 nm. **(B)** Fluorescence reactivity of **2b** (10 μM in DMSO) with various ROS/RNS species upon excitation at 580 nm (F and F_0_ were the fluorescence intensity of the probe in the presence and absence of various ROS/RNS species).

The sensitivity of probe **2b** toward ·OH is investigated by spectrometric titration in DMSO (Figure 3). Fenton reaction between Co(OAc)_2_ and hydrogen peroxide was used to generate ·OH *in situ* in a sample solution. Upon addition of increasing amount of ·OH, the fluorescence intensity at 665 nm increases remarkably with a virtually unchanged peak position upon excitation at 580 nm (Figure 3A) or excitation at 334 nm (Figure S12, ESI). The titration curve of **2b** shows a quick enhancement upon addition small amount of ·OH and then reaches a plateau at 10 equiv of ·OH. The detection limit for ·OH is determined to be 10 μM based on the signal-to-noise ratio of three (Figure S15, ESI). A good linear relationship between the fluorescence response and concentration of ·OH is obtained with a 0.9808 correlation coefficient.

To evaluate the selectivity of **2b** for ·OH, the interference experiments in the presence of several ROS/RNS both in respective and integrated manners are carried out. Almost no change is observed in the fluorescence intensity of **2b** by adding 20 equiv. of ${\text{O}}_{2}^{-}$, 100 equiv. of other ROS/RNS species, such as H_2_O_2_, ClO^−^, TBHP, NO, N${\text{O}}_{2}^{-}$, N${\text{O}}_{3}^{-}$, Vc, GSH, respectively, upon excitation at 580 nm (Figure 3B) or excitation at 334 nm (Figure S13, ESI). Moreover, the fluorescence turn-on response toward ·OH is nearly not interfered in the presence of excess amount of background containing appropriate ROS/RNS species, such as H_2_O_2_, ClO^−^, N${\text{O}}_{2}^{-}$, N${\text{O}}_{3}^{-}$, GSH, and is little interfered in the background containing NO and Vc (Figure S14, ESI). The above results demonstrate that **2b** is a highly selective and sensitive fluorescence turn-on probe for ·OH and its response for ·OH is not interfered in the background containing various ROS/RNS species.

### Sensing Mechanism

**Figure 4:**
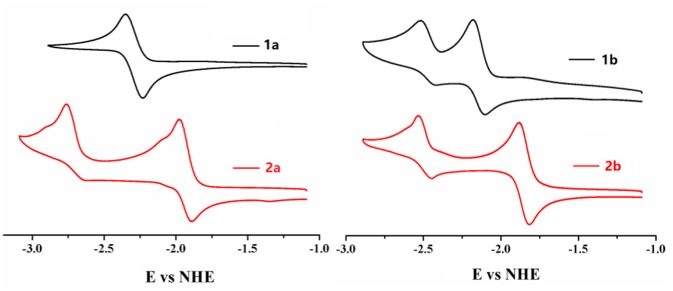
Cyclic voltammograms of compounds **1a, 2a, 1b, 2b** (0.1 mM) in CH_2_Cl_2_ containing 0.1 M NBu_4_PF_6_ (vs. NHE). The ferrocene/ferrocenium (Fc/Fc^+^) couple was used as an external standard.

As the most obvious explanation for the fluorescence turn-on response is that the photo-induced electron transfer (PET) from the lone pair electrons of P(III) atom of triarylphosphine to the BODIPY fluorophore is inhibited upon oxidation by ·OH to form triphenylphosphine oxide P(V)O_2_. To further demonstrate this mechanism, the reaction mode of ·OH with **2b** is investigated by ^31^P NMR spectroscopy (Figure S16, ESI). The ^31^P NMR spectra of **2b** in the presence of different concentrations of ·OH are recorded in DMSO-d_6_ and compared to the spectrum of the free probe. As shown in the ^31^P NMR spectra of **2b**, upon addition of ·OH, the chemical shift of P(III) atom of the triarylphosphine group at −15.73 ppm is significantly down field shifted to 28.89 ppm, which is corresponding to the P(V) of triphenylphosphine oxide, indicating that ·OH oxidize the phosphorus atom of **2b** to form triphenylphosphine oxide. Furthermore, according to the database, the standard oxidation potentials of ·OH, H_2_O_2_, ClO^−^, ${\text{O}}_{2}^{-}$, NO, N${\text{O}}_{2}^{-}$, N${\text{O}}_{2}^{-}$ are 2.8, 1.76, 1.63, 1.59, 0.99, 0.94 V, respectively. Santhanam and Bard reported that the reduction electrode potentials of triphenylphosphine (−2.7 V vs. sce) early in 1967 year (Santhanam and Bard, 1968). Therefore, only the ·OH radical can oxide the triphenylphosphine. Electrochemical properties of **1a**, **2a**, **1b**, **2b** were studied by cyclic voltammetry measured in dry dichloromethane (Figure 4). Probe **2b** consists two reversible reduction potentials at −1.992 and −2.49 V vs. NHE. These results reveal that compound **2b** can be selectively oxidized by ·OH radical without interference in the background containing other RON/ROS.

### The Cell Imaging of ·OH

**Figure 5:**
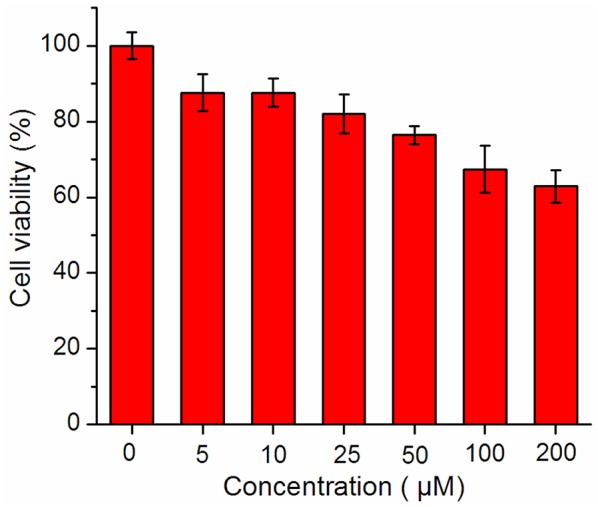
MTT assay of **2b**.

**Figure 6:**
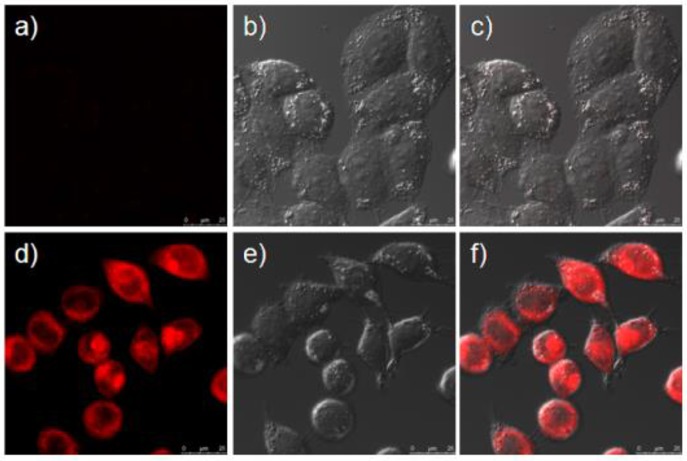
Confocal fluorescence and brightfield images of Hela cells. **(a)** Cells incubated with 10 μM of sensor **2b** for 2 h at 37°C. **(b)** Brightfield image of cells showed in **(a)**. **(c)** One overlay image of **(a,b)**. **(d)** Cells incubated with 10 μM probe at 37°C for 2 h and then treated with phorbol myristate acetate (PMA) for 2 h. **(e)** Brightfield image of cells showed in **(d)**. **(f)** One overlay image of **(d,e)**.

Prior to assess the sensing properties of probe **2b** in a cellular environment, the cytotoxicity of **2b** was evaluated through an MTT assay in HeLa cells. As shown in Figure 5, more than 80% cells are viable after incubation with **2b** over a wide range of concentrations (5–50 μM) for 24 h, indicating that **2b** do not negatively affect the cell viability to HeLa cells. This encouraged us to explore the potential utility of **2b** as a fluorescent probe for ·OH in living cells (Figure 6 and Figure S17 ESI) (Yang et al., 2017). HeLa cells were incubated with 10 μM of **2b** for 30 min at 37 °C and subsequently viewed under confocal microscope upon excitation at 633 nm as control experiments. No intracellular fluorescence was observed (Figure 6a). Then phorbol myristate acetate (PMA) was added in the cells for 2 h, the microscope images exhibited intense intracellular fluorescence (Figure 6d). Bright field measurements, after treatment with both PMA and **2b**, confirmed that the cells are viable throughout the imaging experiments (Figures 6b,c,e,f). Therefore, probe **2b** can clearly be used for intracellular detection of ·OH.

## Conclusions

In summary, a highly selective and sensitive Near IR fluorescent turn-on probe for hydroxyl radical have been designed by utilizing triphenylphosphine as a reaction-site for ·OH in the presence of other reactive oxygen species (ROS) and reactive nitrogen species (RNS). In addition, **2b** can also be applied for bioimaging ·OH in HeLa cells with almost no cytotoxicity, thus demonstrating its application for studying the effect of ·OH in biological systems. These results point the way to a new generation of molecular recognition systems in the NIR window for biological system.

## Data Availability

All datasets generated for this study are included in the manuscript/Supplementary Files.

## Author Contributions

XQ was responsible for carried out the experiments and performing spectroscopic measurements. WS was responsible for carrying out the cell imaging experiments. ZS was responsible for designing the project and revising the manuscript.

### Conflict of Interest Statement

The authors declare that the research was conducted in the absence of any commercial or financial relationships that could be construed as a potential conflict of interest.
