# Supplementary material for: A Highly Selective NIR Fluorescent Turn-on Probe for Hydroxyl Radical and Its Application in Living Cell Images
Source: Front Chem. 2019 Aug 28;7:598. doi: 10.3389/fchem.2019.00598 (PMC6724658; doi:10.3389/fchem.2019.00598)
Supplement: Supplementary file 1 [file Data_Sheet_1.pdf]

Supplementary Material

A Highly Selective NIR Fluorescent Turn-on Probe for Hydroxyl Radical and Its Application in Living Cell Images

Xingyu Qu[1,2], Wenting Song[1], Zhen Shen [1*],

[1] State Key Laboratory of Coordination Chemistry, Nanjing National Laboratory of Microstructures, Nanjing 210093, P. R. China.

[2] Department of Chemistry and Chemical Engineering, Jinzhong University, Jinzhong, Shanxi, 030619, P. R. China

*** Correspondence:**

Zhen Shen
zshen@nju.edu.cn

Contents:

Figure S1 ^{1}H NMR spectra of compound **2a**.

Figure S2 ^{1}H-^{1}H COSY NMR spectrum of compound **2a**.

Figure S3 ^{1}H NMR spectra of compound **2b**.

Figure S4 ^{1}H-^{1}H COSY NMR spectrum of compound **2b**.

Figure S5 ^{13}C NMR spectrum of compound **2b**.

Figure S6 ^{1}H NMR spectra of compound **ox-2a**.

Figure S7 ^{1}H-^{1}H COSY NMR spectrum of compound **ox-2a**

Figure S8 ^{13}C NMR spectrum of compound **ox-2a**.

Figure S9 MALDI-TOF MS spectrum of compound **2a.**

Figure S10 MALDI-TOF MS spectrum of compound **2b.**

Figure S11 MALDI-TOF MS spectrum of compound **ox-2a**.

Figure S12 Changes in the fluorescence spectrum of **2b** as the •OH concentration is increased.

Figure S13 The fluorescence intensity of **2b** is observed at 665 nm upon addition of various RON/ROS.

Figure S14 Fluorescence intensity ratio histograms of **2b** dispersed in DMSO with the adding of corresponding radicals and subsequent adding of •OH.

Figure S15 Fluorescence intensity of probe **2b** after reacting with •OH as a function of •OH concentration.

Figure S16 ^{31}P NMR of **2b** with different concentrations of •OH in DMSO-d_6.

Figure S17 Confocal fluorescence and brightfield images of Hela cells.

Table S1 Crystal data and structure refinement for **ox-2a.**

Table S2 Optical properties of **2a**, **2b** and **ox-2a** in various solvents at 298 K.

Supplementary Figures and Table

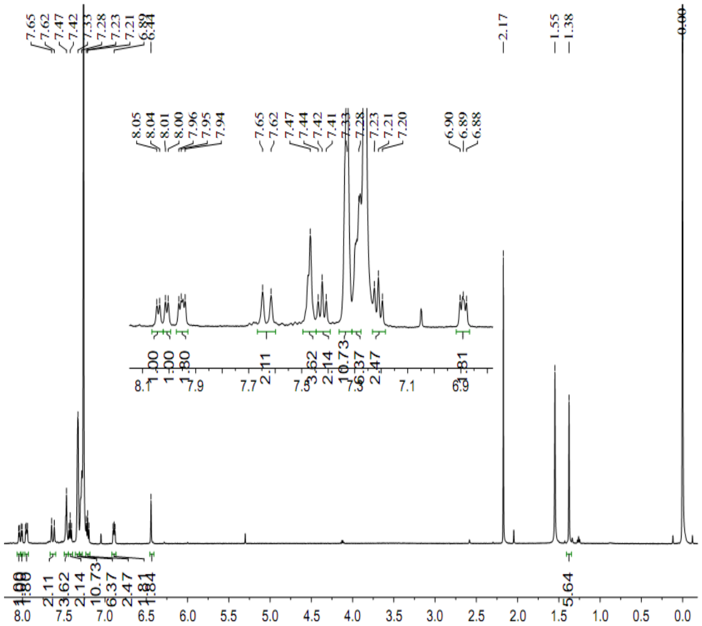

Figure S1 ^{1}H NMR spectrum of compound **2a** ($CDCl_3$, 298 K)

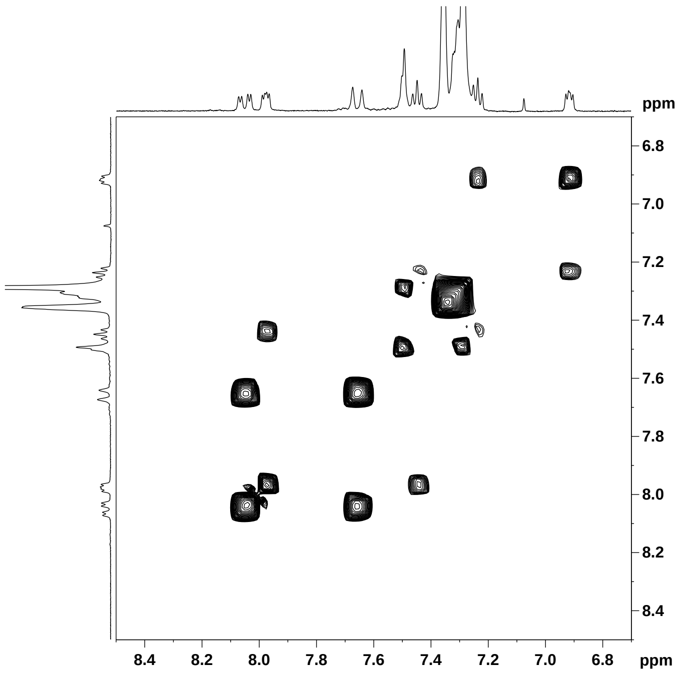

Figure S2 ^{1}H-^{1}H COSY spectrum of compound **2a** ($CDCl_3$, 298 K)

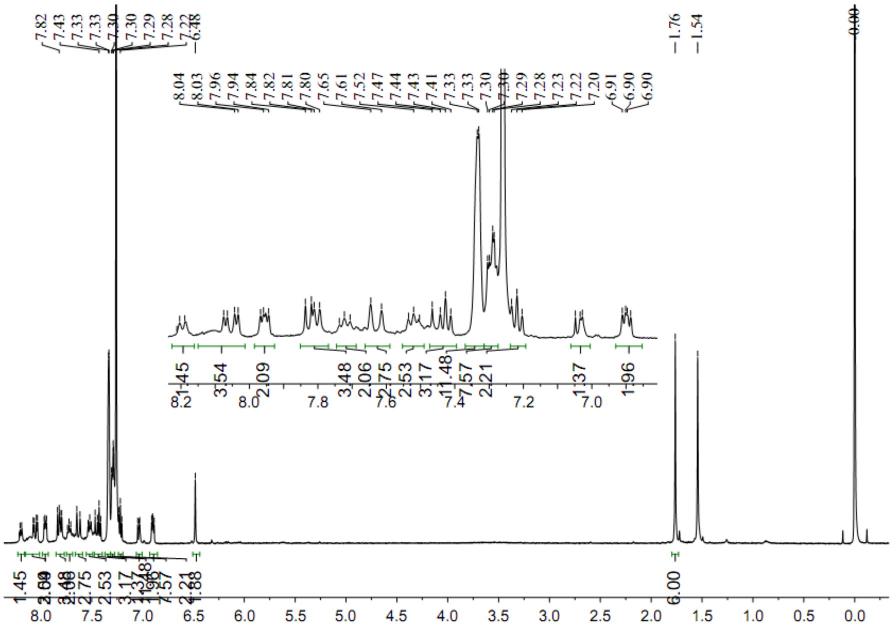

Figure S3. ^{1}H NMR spectrum of compound **2b** ($CDCl_3$, 298 K)

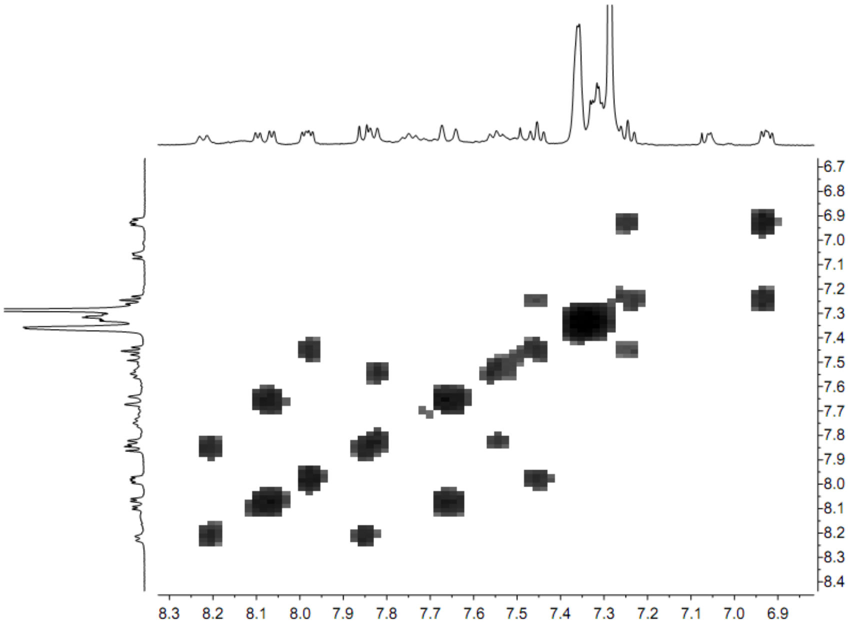

Figure S4. ^{1}H-^{1}H COSY spectrum of compound **2b** ($CDCl_3$, 298 K).

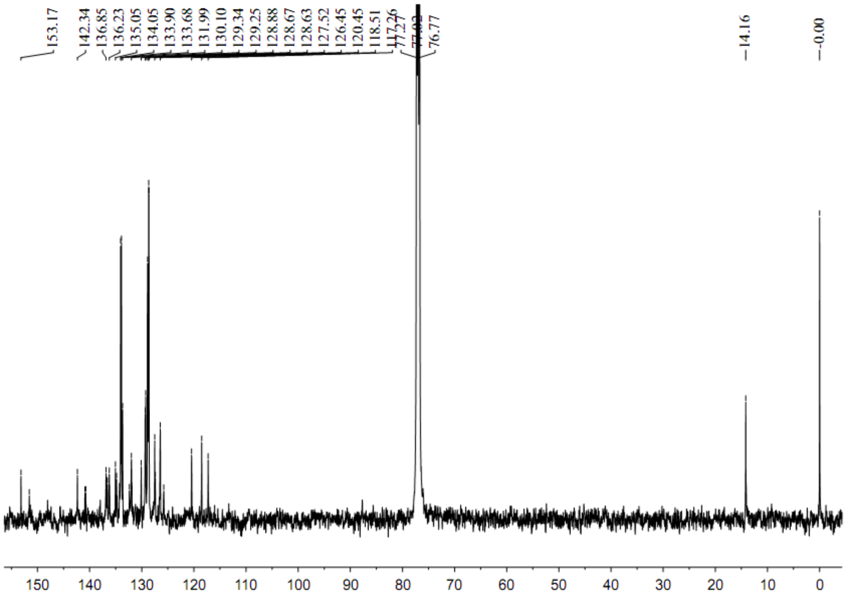

Figure S5. ^{13}C NMR spectrum of compound **2b** ($CDCl_3$, 298 K).

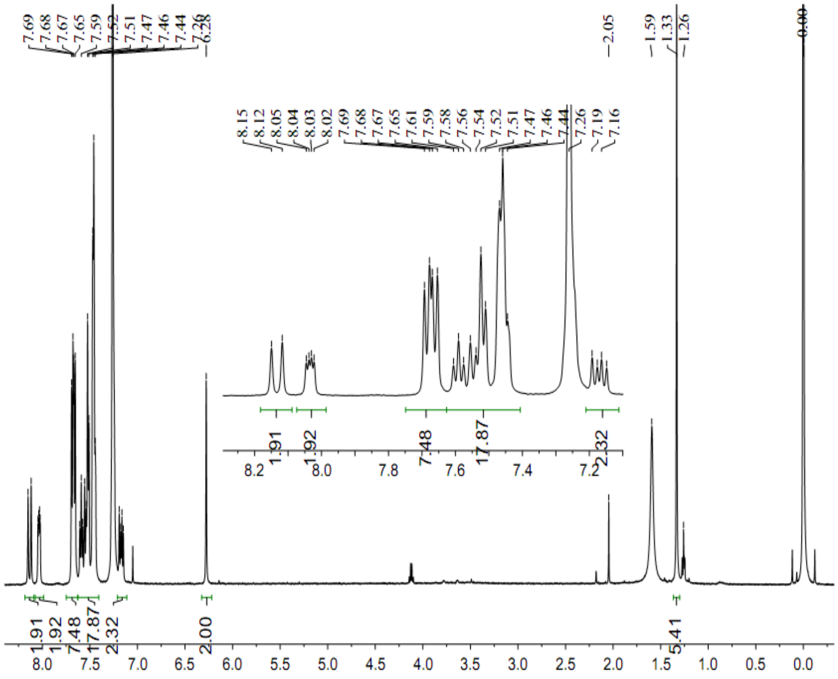

Figure S6. ^{1}H NMR spectrum of compound **ox-2a** ($CDCl_3$, 298 K)

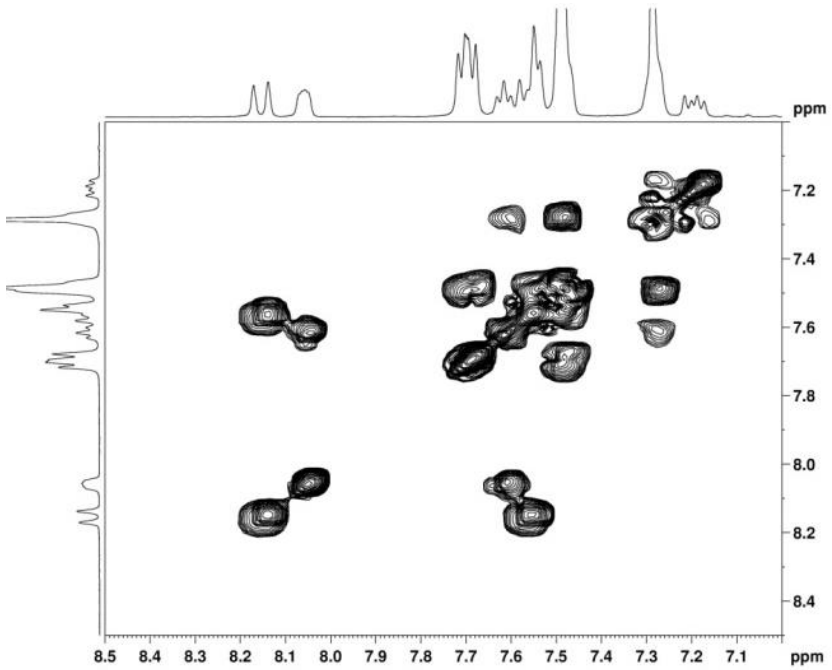

Figure S7. ^{1}H-^{1}HCOSY spectrum of compound **ox-2a** ($CDCl_3$, 298 K)

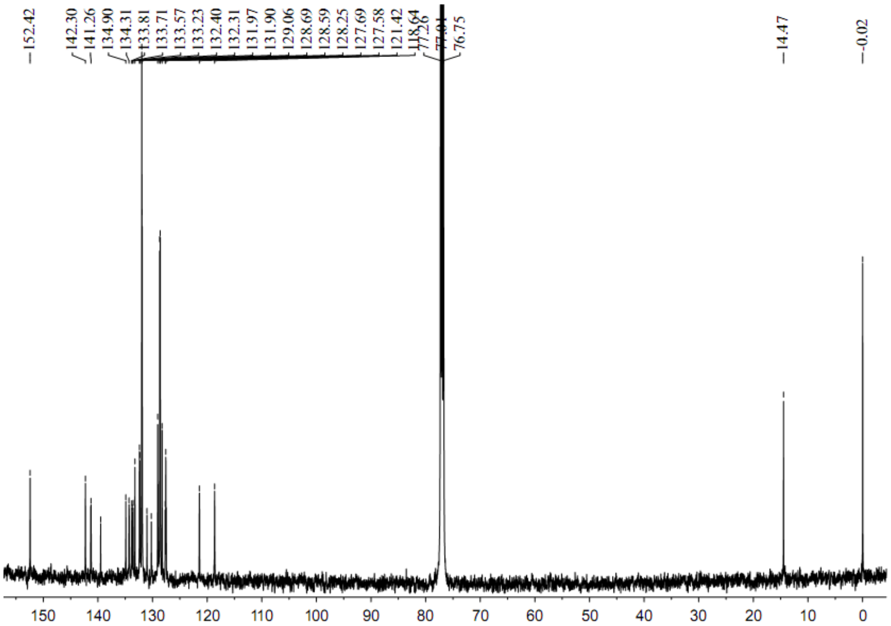

Figure S8. ^{13}C NMR spectrum of compound **ox-2a** ($CDCl_3$, 298 K).

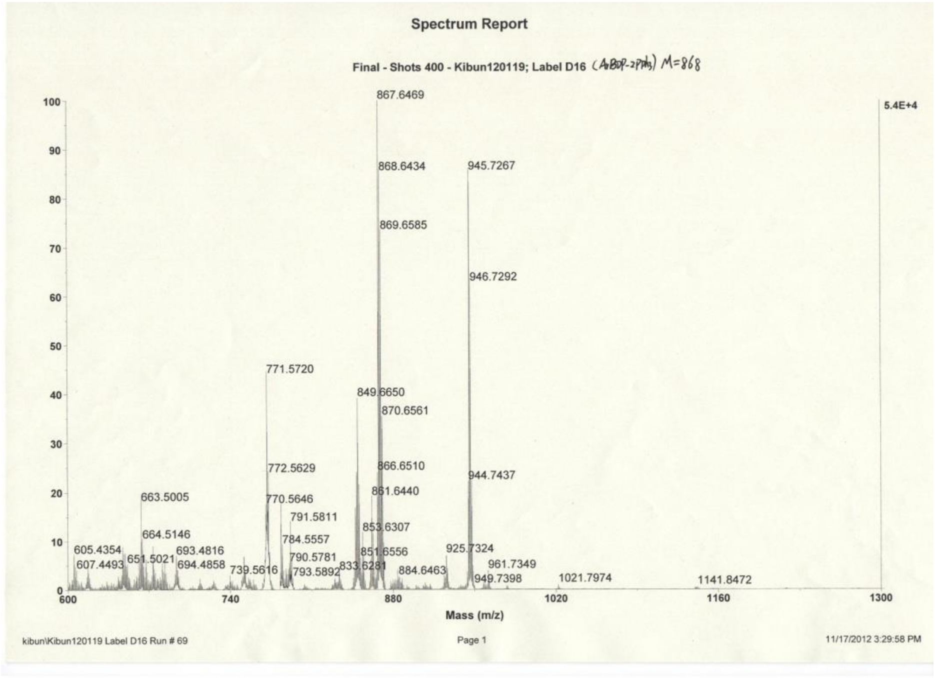

Figure S9. MALDI−TOF MS spectrum of compound **2a** .

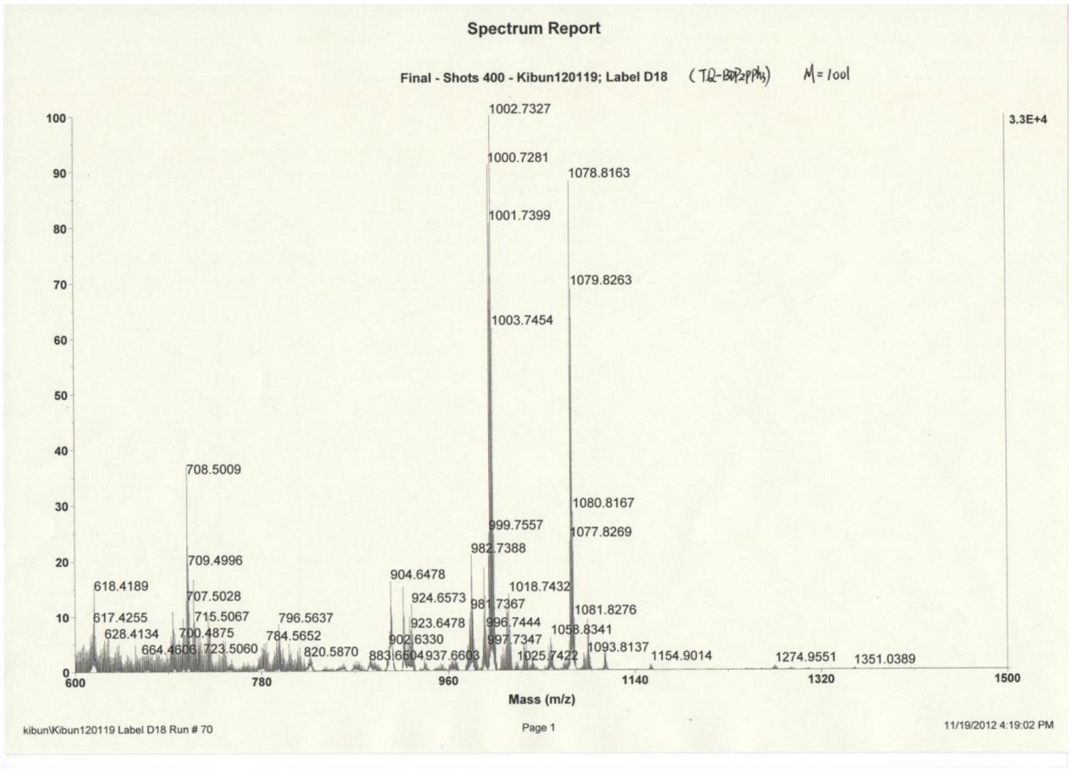

Figure S10. MALDI−TOF MS spectrum of compound **2b** .

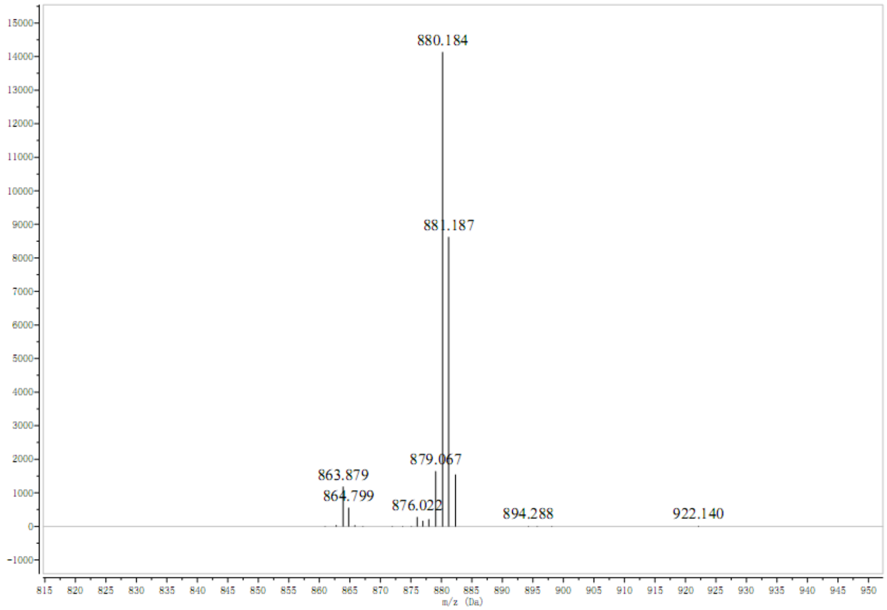

Figure S11. MALDI−TOF MS spectrum of compound **ox-2a**.

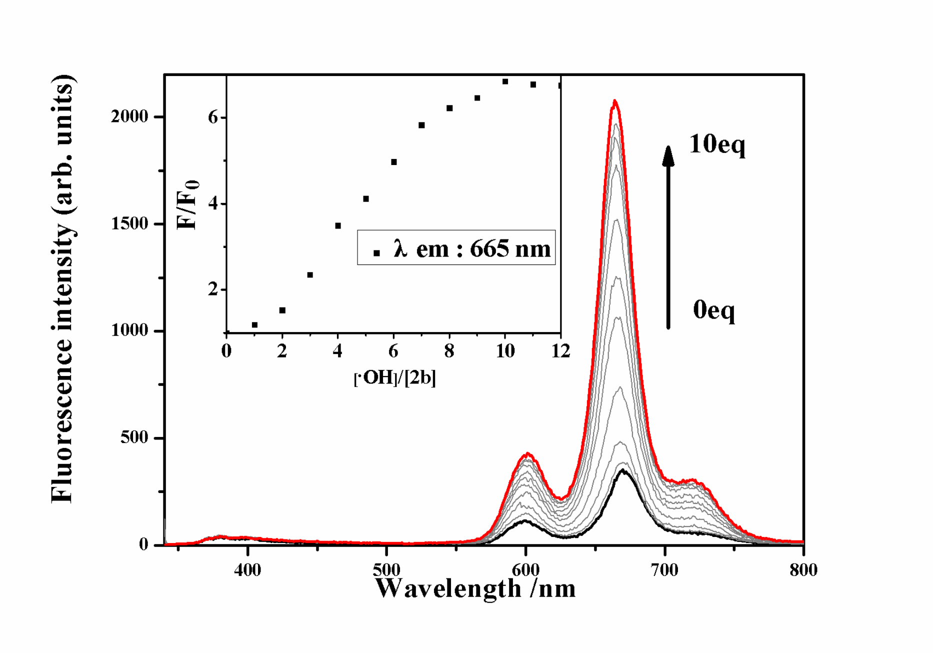

Figure S12. Changes in the fluorescence spectrum of **2b** (10 μM in DMSO) as the •OH concentration is increased upon excitation at 334 nm. The inset shows that the emission intensity at 665 nm changes as a function of the •OH concentration

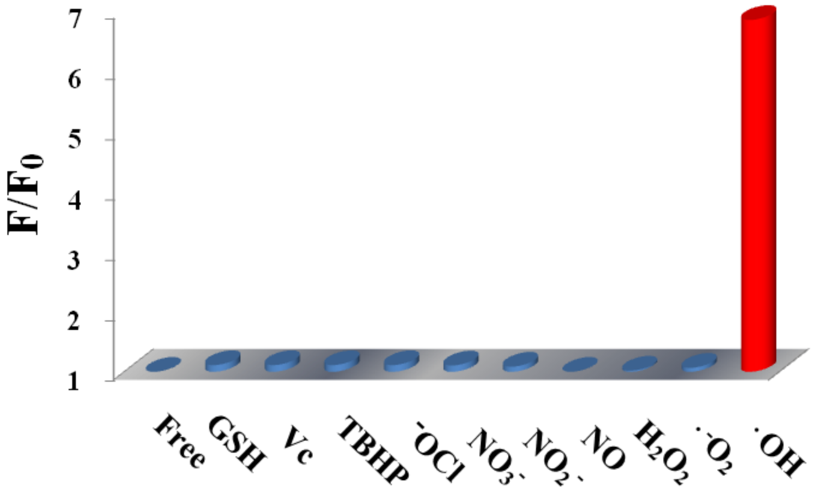

Figure S13. The fluorescence intensity of **2b** is observed at 665 nm upon addition of various RON/ROS upon excitation at 334 nm.

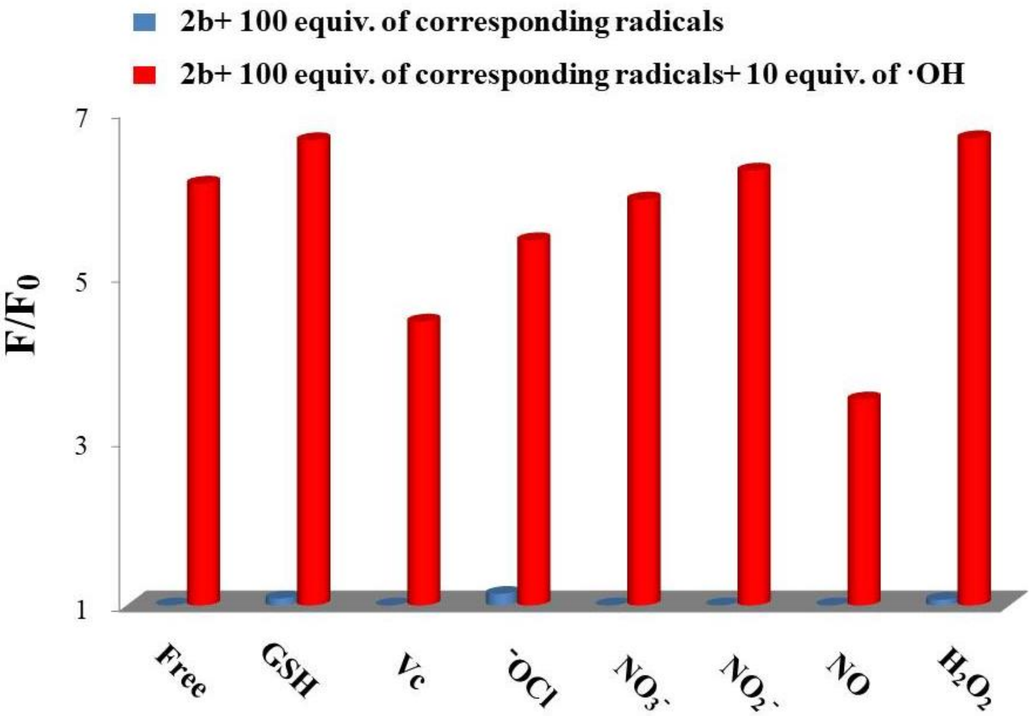

Figure S14. Fluorescence intensity ratio histograms of **2b** dispersed in DMSO with the adding of corresponding radicals (red) and subsequent adding of 10 equiv. of •OH (blue).

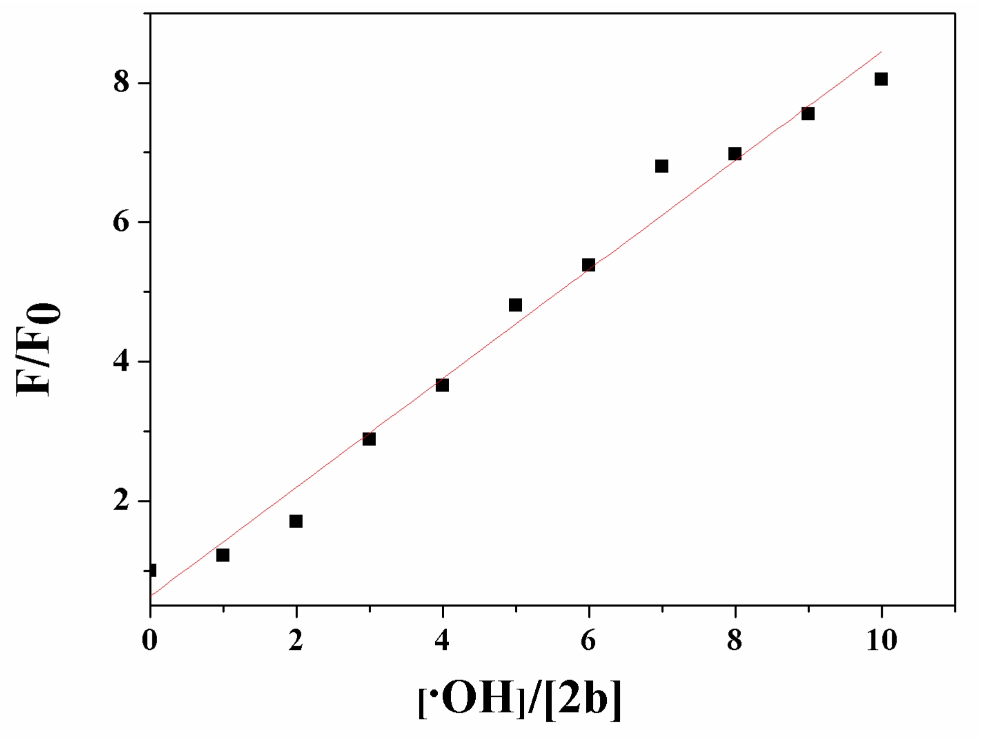

Figure S15. Fluorescence intensity of probe **2b** after reacting with ·OH as a function of ·OH concentration

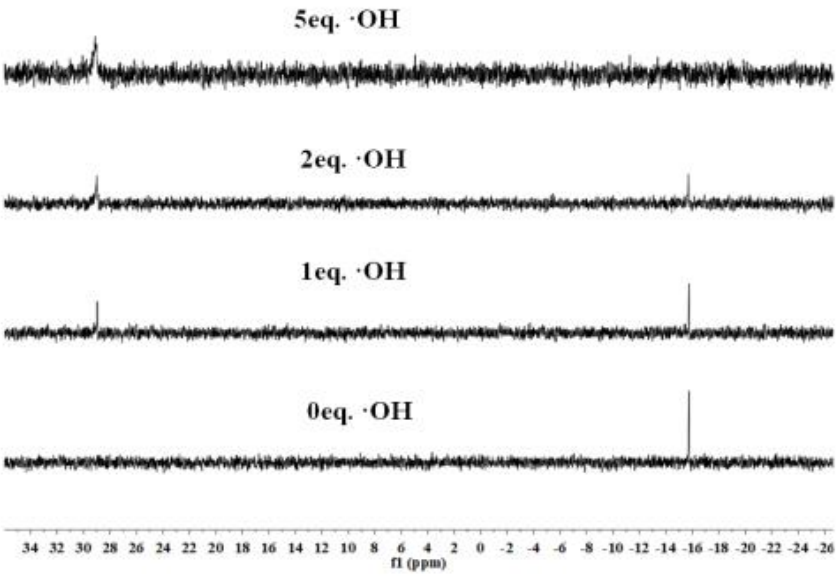

Figure S16. ^{31}P NMR of **2b** with different concentrations of ·OH in DMSO-d_6.

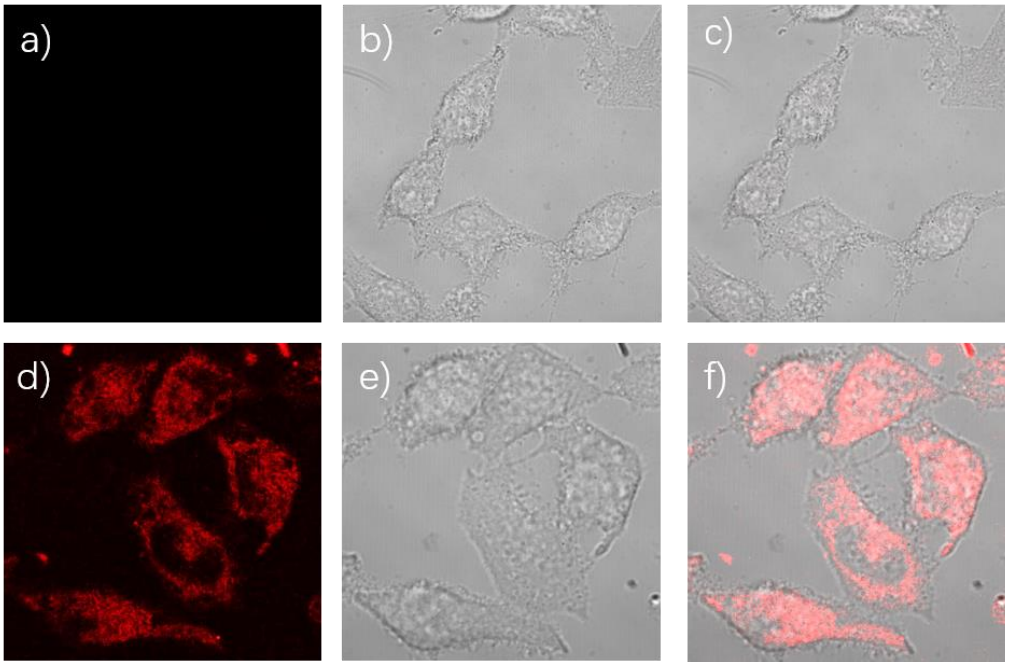

Figure S17. Confocal fluorescence and brightfield images of Hela cells. (a) Cells incubated with 10 μM of **2b** for 30 min at 37 °C. (b) Brightfield image of cells showed in panel a. (c) One overlay image of (a) and (b). (d) Cells incubated with 10 μM **2b** and H_2O_2 (1mM) at 37 ℃ for 30min. (e) Brightfield image of cells showed in panel d. (f) One overlay image of (d) and (e).

Table S1. Crystal data and structure refinement for **ox-2a**

Parameters name	Data	Parameters name	Data
Empirical formula	$C_{57}H_{45}BF_2N_2O_2P_2$	Density (calc.) Mg/m3	1.276
Formula weight	900.3	Absorption coefficient (mm ^{1})	0.146
Crystal size (mm^3)	0.18×0.15×0.11	Temperature (K)	123(2)
Crystal system	Monoclinic	F(000)	1880
Space group	*P2(1)/c*	Goodness of fit on F^{-2}	1.028
Z	4	Max. and min. absorption	0.9841 and 0.9741
a /Å	16.460(7)	range for data collection (°)	1.93 to 28.37
b /Å	12.384(4)	Index ranges	-20≤ h ≤20,<br>-15≤ k ≤7,<br>-28≤ l ≤28
c / Å	23.223(10)	Total reflections	4617
α / (deg)	90	Independent reflections	2686 [$R_{(int)}$ = 0.1167]
/ (deg)	97.818(9)	Parameters	277
γ / (deg)	90	Final R indices [I > 2]	R_1 = 0.1051<br>wR_2 = 0.1357
Volume / Å^3	4690(3)	R indices (all data)	R_1 = 0.0495<br>wR_2 = 0.1176

Table S2. Optical properties of **2a**, **2b** and **ox-2a** in various solvents at 298 K

compound	solvent	λ_{abs}^{max}(nm)	λ_{em}^{ex334} (nm)	$\lambda_{em}^{acceptor}$ (nm)	Φ_f^{ex334}	$\Phi_f^{accptor}$	ETE
	Hexane	628, 578, 345	—	644	—	0.77	—
	CH_2Cl_2	633, 577, 350	—	650	—	0.72	—
**2a**	CH_3OH	625, 576, 345	—	641	—	0.57	—
	CH_3CN	624, 574, 344	—	642	—	0.25	—
	DMSO	631, 582	—	650	—	0.20	—
	Hexane	645, 591, 352	660	660	0.46	0.29	157%
	CH_2Cl_2	650, 594, 356	667	668	0.54	0.32	169%
**2b**	CH_3OH	640, 590, 353	659	658	0.33	0.22	150%
	CH_3CN	639, 590, 352	659	659	0.11	0.8	138%
	DMSO	650, 594	668	666	0.06	0.08	75%
	Hexane	625, 581, 350	—	641	—	0.85	—
	CH_2Cl_2	630, 584, 355	—	646	—	0.74	—
**ox-2a**	CH_3OH	624, 574, 353	—	638	—	0.81	—
	CH_3CN	622, 576, 350	—	638	—	0.78	—
	DMSO	628, 581	—	645	—	0.69	—
